# Public perception of the appropriateness of COVID-19 management strategies and level of disturbances in daily activities: A focus on educational level

**DOI:** 10.1371/journal.pone.0287143

**Published:** 2023-06-09

**Authors:** Yeong Jun Ju, Woorim Kim, Soon Young Lee

**Affiliations:** 1 Department of Preventive Medicine and Public Health, Ajou University School of Medicine, Suwon-si, Gyeonggi-do, Republic of Korea; 2 Division of Cancer Control & Policy, National Cancer Control Institute, National Cancer Center, Goyang-si, Gyeonggi-do, Republic of Korea; Centre for Demographic Studies, SPAIN

## Abstract

**Background:**

This study investigated the association between public perception of the appropriateness of management strategies implemented during the COVID-19 pandemic and the level of disturbances in daily activities reported by the general population.

**Methods:**

This cross-sectional study used Korea Community Health Survey conducted from August to November 2020. Public perception of COVID-19 management strategies included those implemented by the government (central, city or provincial, and administrative districts), the mass media, regional medical institutions, and neighbors. The subjective level of disturbances in daily activities was measured using a 0–100 numeric rating scale developed by Korea Disease Control and Prevention Agency. Multivariable linear regression analysis was used. A subgroup analysis was conducted based on education level.

**Results:**

The present study analyzed 211,353 participants. Compared to individuals who perceived that the management strategies implemented during the pandemic was ‘highly appropriate,’ those who reported ‘mediocre appropriateness’ (β: -1.96, p-value: <0.001) or ‘low appropriateness’ (β: -3.60, p-value: 0.010) reported higher levels of subjective disturbances. The appropriateness of measures implemented by the mass media was associated with levels of subjective disturbances felt by individuals of lower education with statistical significance, whereas that applied by the mass media and the government were important in those with higher education.

**Conclusions:**

The findings suggest the importance of public perception of management strategies when implementing containment policies and minimizing its disturbances on daily lives is essential.

## Introduction

The coronavirus disease 2019 (COVID-19) was declared a pandemic by the World Health Organization (WHO) in 2020 [1]. The number of infected individuals and related mortality has been increasing due to its rapid rate of transmission worldwide [2]. Naturally, the pandemic has widely affected the lives of numerous individuals, in which its direct impact includes the lowering of psychological well-being and quality of life [3]. The outbreak has also exerted many indirect effects, due to social distancing and quarantine measures implemented by the government to confine and manage cases [4]. The government of South Korea also implemented a “distancing in daily life” initiative along with rapid testing and isolation strategies to respond to the outbreak [4, 5].

Although social distancing is an effective strategy to reduce the spread of COVID-19, such measures have led to difficulties in physically contacting close contacts, an increase in working from home, and a rise in uncertainty about future employment and income due to job loss [3]. Furthermore, these circumstances, including the closing of schools and shops, the ban of public events, and the promotion of working from home, have inevitably affected the daily activities of several individuals in many different aspects [6]. For instance, changes in economic activity, work, sleep, physical activity, and socialization have been reported [7]. Routinized behaviors are known to provide a sense of purpose and meaning in life, which is the essence of wellbeing [8]. As such, alterations in daily life patterns can exert a negative influence on wellbeing and increase psychological problems, such as depression, anxiety, and emotional fatigue [9]. Impact on social life may also negatively affect subjective wellbeing and life satisfaction [10]. At the same time, the pandemic may have also exerted some positive effects, such as increased utilization of the internet and work flexibility [11, 12]. Considering the impact of COVID-19 on the daily lives of individuals, a need exists to investigated the associated factors.

Many aspects of a pandemic are mediated, explained, or experienced through governmental institutions and mass media, which implies that public perception of these institutions is involved with how individuals interact with a crisis [13]. Previous literature has reported the potential impact of public perception or trust during the COVID-19 outbreak on the life of individuals. A study in Germany revealed that individuals with a low level of trust in government institutions or the media pre-crisis report a larger decrease of life satisfaction [14]. Trust in COVID-19 related regulations implemented by governmental and social institutions was also related to overall frustration in university students [15]. Also, as dissemination of reliable information in a timely manner is essential to motivate the public to conform with restrictive measures during times of crisis, securing channels of communication between the government and society have been found important during the pandemic [16].

As literature suggest, public perception of the appropriateness of management strategies implemented during a pandemic may be related to the subjective level of disturbances sensed by an individual. Public trust may be associated with the level of daily life disruptions during a crisis by affecting individuals’ willingness to adapt and conform to public policy interventions [17]. Adherence to government guidelines during a pandemic is also dependent on trust in the government and its institutions, which implicates its importance in terms of societal response [18]. Perception of the mass media is also an influential factor because it allows fast and extensive reach regarding public health communication while also providing social connectedness [19]. Disturbances in social relationships and medical services during a pandemic are impacted by communication as it delivers information from both authorities and informal social networks [20, 21].

Despite its potential importance, studies on the association between public perception of the appropriateness of COVID-19 management strategies and the level of disturbances in daily activities reported during the outbreak in Korea are deficient. Hence, this study aimed to investigate whether individuals who perceived the implemented COVID-19 management strategies as being positive report lower levels of disturbances in daily activities. Analysis was also conducted by how the public perceived the appropriateness of each type of COVID-19 measure, which included that implemented by central government, the city or provincial government, the administrative district government, the mass media, regional medical institutions, and neighbors and coworkers. This was because measures were implemented or distributed at different levels during the outbreak. Subgroup analysis was conducted based on education level because education is a well-known constituent of socioeconomic status and different socioeconomic groups are known to respond differently to life satisfaction [22]. Education also has an effect on institutional trust, with the relationship often being positive in cleaner countries and negative in corrupt societies [23]. As such, this study additionally explored how education level interplays in the stated relationship as different educational groups are likely to exhibit different patterns. The hypothesis was that public perception of the coping strategies implemented during the pandemic as being appropriate will be associated with lower levels of disturbances in daily activities reported, particularly in individuals with higher levels of education.

## Materials and methods

### Data and study population

This study used raw data from the 2020 Korea Community Health Survey (KCHS) conducted by the Korea Centers for Disease Control and Prevention Agency (KDCA). The KCHS is a cross-sectional survey, with a study population drawn from multistage, stratified area probability samples of civilian, non-institutionalized Korean households categorized according to geographic area, age, and sex. The survey is conducted annually and collects data through in-person (one-on-one) interviews. As the population sample is extracted from national survey data, it is considered representative of the Korean population [24].

This study included individuals aged ≥19 years. From an initial total of 229,269 potential participants, those with missing data on the relevant variables were excluded. This left a total of 211,353 participants eligible for inclusion in the present study (S1 Fig).

### Outcome variable

The outcome variable of this study was the subjective level of disturbances in daily activities reported by the study participants. This variable was measured using a 0–100 numeric rating scale developed by the KDCA to measure the impact of the COVID-19 in Korea and report the results as national statistics. This variable was measured based on the following question: “Assuming that a score of 100 implies that your daily life is the same as before the COVID-19 outbreak whereas a score of 0 implies a complete level of disturbance, what is your current status?” Participants could respond on a ten-point unit. A lower score implies a higher level of disturbance. The subjective level of disturbances in daily activities was successfully evaluated using this question in a previous study [25].

### Independent variables

The main variable of this study was public perception of the appropriateness of management strategies during the COVID-19 pandemic, measured using the question: “Do you think that the government or the following institutions have implemented appropriate COVID-19 coping strategies?” The question was inquired for the central government (including the Ministry of Health and Welfare and KCDA), the city or provincial government, the administrative district government, the mass media, regional medical institutions, and neighbors and coworkers. Available responses were “very appropriate,” “appropriate,” “mediocre,” “inappropriate,” and “very inappropriate.” Individuals who responded that the coping strategies were appropriate were classified into the “yes” category and vice versa. The questions were inquired separately and in composite. In the composite analysis, the responses for the different types of measures inquired were summed up and categorized into “Appropriate” (5 to 6), “Mediocre” (3 to 4), and “Not appropriate” (0 to 2) groups to measure how positively individuals perceived COVID-19 management strategies in whole.

Various demographic, socioeconomic, and health-related variables were included as covariates for the analysis. They were sex (male or female), age (19–29, 30–39, 40–49, 50–59, 60–69, or 70+ years), education level (uneducated, elementary school, middle school, high school, or college and above), income (quartiles), job classification (professional or administrative position, office work, sales and service, agriculture and fishery, blue-collar work or simple labor, or unemployed), household composition (one-, two-, or three-generation household), area of residence (urban or rural), monthly drinking status (yes or no), smoking status (yes or no), depressive symptoms (yes or no), perceived stress (yes or no), and subjective health status (poor or fair).

### Statistical analysis

The general characteristics of the study population were examined by t-test, and analysis of variance was used to compare the means and standard deviations in subjective level of disturbances in daily activities by characteristics. Additionally, Cohen’s *d*, an effect-size measure, was calculated (S1 Table). Multivariable linear regression analysis was used to examine the association between public perception of implemented COVID-19 management strategies and subjective levels of disturbances in daily activities. Analyses were also performed separately by the six different kinds of management strategies. Subgroup analysis was conducted based on education level. All p-values were considered two-sided and significant at p<0.05. All analyses were conducted using the SAS software, version 9.4 (SAS Institute, Cary, NC, USA).

## Results

The general characteristics of 211,353 study population are shown in Table 1. About 61.6% of the study population perceived the implemented COVID-19 management strategies as being “appropriate” (5 to 6), 16.1% as “mediocre” (3 to 4), and 22.3% as “not appropriate” (0 to 2). The mean level of disturbances in daily activities reported by the participants was 55.33 ± 22.96. Increased scores, inferring low levels of disturbances in daily activities, were found in individuals who viewed the introduced coping strategies as being appropriate.

**Table 1:** General characteristics of subjects.

Variables	Total		Level of disturbances in daily activities*	*P*-Value
	N	%	Mean ± S.D	
**Public perception of management strategies**				
Not appropriate (0~2)	47,177	22.3	51.68 ± 23.64	< .001
Mediocre (3~4)	34,087	16.1	53.46 ± 22.81	
Appropriate (5~6)	130,089	61.6	57.14 ± 22.55	
**Sex**				
Male	95,868	45.4	56.69 ± 22.63	< .001
Female	115,485	54.6	54.19 ± 23.18	
**Age**				
19–29	24,297	11.5	53.13 ± 21.52	< .001
30–39	23,611	11.2	50.78 ± 22.01	
40–49	33,400	15.8	53.03 ± 21.39	
50–59	40,942	19.4	54.52 ± 22.46	
60–69	41,434	19.6	55.69 ± 23.61	
70+	47,669	22.6	60.69 ± 24.04	
**Educational level**				
Uneducated	18,579	8.8	62.65 ± 24.34	< .001
Elementary school	30,602	14.5	58.53 ± 24.14	
Middle school	23,038	10.9	55.95 ± 23.68	
High school	72,159	34.1	53.98 ± 22.59	
College and above	66,975	31.7	53.07 ± 21.54	
**Income**				
Q1 (Low)	52,175	24.7	58.61 ± 24.90	< .001
Q2	47,883	22.7	55.09 ± 23.33	
Q3	52,184	24.7	53.99 ± 22.08	
Q4 (High)	59,111	28.0	53.80 ± 21.28	
**Job classification**				
Professional or administrative position	21,630	10.2	53.35 ± 21.25	< .001
Office work	18,623	8.8	54.63 ± 20.33	
Sales and service	27,028	12.8	51.99 ± 22.40	
Agriculture and fishery	20,897	9.9	60.79 ± 23.41	
Blue collar work or simple labor	39,964	18.9	56.58 ± 22.31	
Unemployed	83,211	39.4	55.11 ± 24.01	
**Household composition**				
1 generation	100,156	47.4	57.07 ± 23.72	< .001
2 generation	97,316	46.0	53.60 ± 22.09	
3 generation	13,881	6.6	54.83 **±** 22.48	
**Area of residence**				
Rural	92,499	43.8	58.17 ± 23.59	< .001
Urban	118,854	56.2	53.11 ± 22.21	
**Monthly drinking status**				
No	116,440	55.1	56.16 ± 23.54	< .001
Yes	94,913	44.9	54.31 ± 22.19	
**Smoking status**				
No	177,505	84.0	55.34 ± 22.88	0.510
Yes	33,848	16.0	55.25 ± 23.39	
**Depressive symptoms**				
No (PHQ-9 < 10)	205,539	97.3	55.47 ± 22.81	< .001
Yes (PHQ-9 ≥ 10)	5,814	2.8	50.30 ± 27.18	
**Perceived stress**				
No	164,836	78.0	56.78 ± 22.56	< .001
Yes	46,517	22.0	50.19 ± 23.62	
**Subjective health status**				
Poor	109,471	51.8	55.23 ± 23.22	0.050
Fair	101,882	48.2	55.43 ± 22.68	
**Total**	211,353	100.0	55.33 ± 22.96	

* Lower score implies higher levels of disturbances

The results of the regression analysis on the association between levels of disturbances in daily activities and perceived appropriateness of COVID-19 management strategies are presented in Table 2. Compared to individuals who viewed the strategies as being “appropriate”, those who perceived them as being “mediocre” (β -1.96, p-value <0.001) or “not appropriate” (β -3.60, p-value 0.010) showed increased levels of disturbances in daily activities.

**Table 2:** Results of the multivariable linear regression analysis.

Variables	Level of disturbances in daily activities*		
	Adjusted-β	S.E.	*P*-value
**Public perception of management strategies**			
Appropriate (5~6)	Ref.		
Mediocre (3~4)	-1.96	0.18	< .001
Not appropriate (0~2)	-3.60	0.17	0.010
**Sex**			
Male	Ref.		
Female	-3.65	0.15	< .001
**Age**			
19~29	Ref.		
30~39	-2.82	0.26	< .001
40~49	-1.40	0.23	< .001
50~59	-0.40	0.23	0.072
60~69	-0.51	0.27	0.060
70+	2.17	0.32	< .001
**Education level**			
Uneducated	Ref.		
Elementary school	-3.21	0.37	< .001
Middle school	-4.45	0.41	< .001
High school	-4.34	0.40	< .001
College and above	-4.69	0.41	< .001
**Income**			
Q1 (Low)	Ref.		
Q2	-1.08	0.29	< .001
Q3	-0.76	0.32	0.017
Q4 (High)	-0.40	0.33	0.229
**Job classification**			
Professional or administrative position	Ref.		
Office work	1.31	0.25	< .001
Sales and service	-1.79	0.27	< .001
Agriculture and fishery	0.96	0.36	0.009
Blue collar work or simple labor	0.17	0.25	0.494
Unemployed	-2.60	0.24	< .001
**Household composition**			
1 generation	Ref.		
2 generation	-0.32	0.18	0.078
3 generation	-0.88	0.33	0.008
**Area of residence**			
Rural	Ref.		
Urban	-1.87	0.20	< .001
**Monthly drinking status**			
No	Ref.		
Yes	-0.87	0.15	< .001
**Smoking status**			
No	Ref.		
Yes	-0.26	0.20	0.193
**Depressive symptoms**			
No (PHQ-9 < 10)	Ref.		
Yes (PHQ-9 ≥ 10)	-2.53	0.47	< .001
**Perceived stress**			
No	Ref.		
Yes	-4.99	0.17	< .001
**Subjective health status**			
Poor	Ref.		
Fair	0.87	0.14	< .001

* Lower score implies higher levels of disturbances

Apart from public perception of management strategies, Table 2 additionally reveal other factors associated with the level of disturbances in daily activities experienced by individuals. Specifically, females and those aged between 20 to 49 years tended to report higher levels of disturbances, whereas the elderly aged 70 years or above showed a reverse relationship. Individuals with a higher education also reported increased levels of disturbances, in addition to those residing in urban areas or belonging to households composed of three generations. Regarding occupation, higher levels of disturbances were shown in those in sales and service workers or the unemployed than those in professional or administrative positions, but a reversed association was found in those in office work or agriculture and fishery. Individuals with depressive symptoms or perceived stress also showed escalated levels of disturbances.

**Fig 1:**
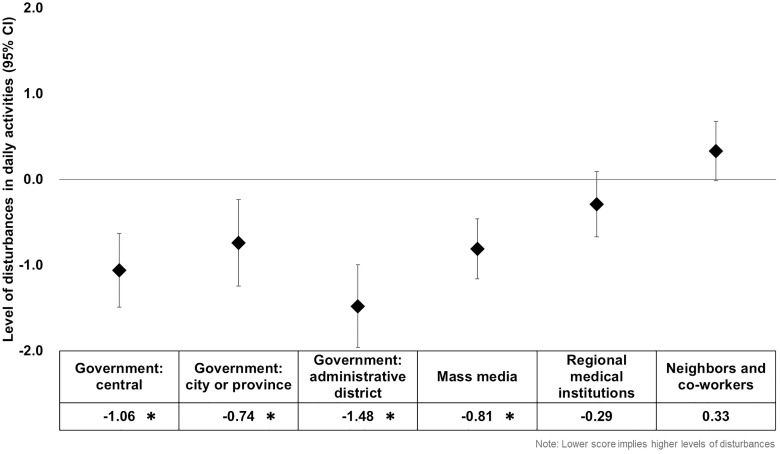
Results of the multivariable linear regression analysis by public perception on the type of COVID-19 management strategies. * Indicates a value of *p* < 0.05.

The results of the analysis considering each type of COVID-19 measure separately are presented in Fig 1. A higher level of disturbances in daily activities was found in individuals who viewed those strategies implemented by the central government (β -1.06, p-value <0.001), the city or province (β -0.74, p-value = 0.004), the administrative district government (β -1.48, p-value <0.001), and the mass media (β -0.81, p-value <0.001) as being inappropriate.

**Fig 2:**
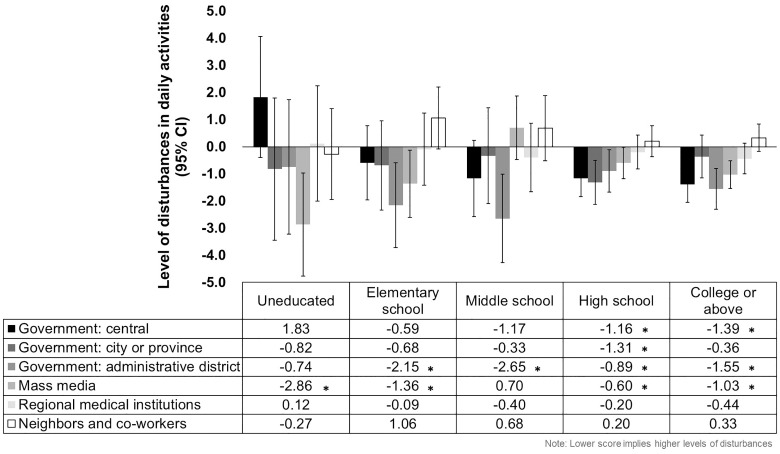
Results of subgroup analysis of the multivariable linear regression analysis of the association between public perception of COVID-19 management strategies and levels of disturbances in daily activities by education level. * Indicates a value of *p* < 0.05.

The results of the subgroup analysis are shown in Fig 2. The level of disturbances in daily activities was associated with public perception of the appropriateness COVID-19 management strategies related to the mass media in the “uneducated” (β -2.86, p-value 0.003) “elementary school” (β -1.36, p-value 0.031) groups. Statistical significance was found with that of the administrative government in the “elementary school” (β -2.15, p-value 0.007) and “middle school” (β -2.65, p-value 0.002) groups. The “high school” (central government: β -1.16, p-value 0.001; city or provincial government: β -1.31, p-value 0.002; administrative government: β -0.89, p-value 0.026; mass media: β -0.60, p-value 0.043) and “college and above” (central government: β -1.39, p-value <0.001; administrative government: β -1.55, p-value <0.001; mass media: β -1.03, p-value <0.001) groups generally showed a correlation between the level of disturbances in daily activity and public perception of strategies by the mass media and all levels of the government.

## Discussion

The findings of this study reveal that individuals who perceived that management strategies implemented during the COVID-19 outbreak as being appropriate reported lower levels of disturbances in daily activities. In other words, the higher the trust in institutions, indirectly measured based on appropriateness in this study, the smaller the disturbances individuals experienced due to the pandemic. Specifically, appropriateness of strategies introduced by the central government, the city or provincial government, the administrative district government, and the mass media were related with lower levels of disturbances reported by individuals. Interestingly, appropriateness of strategies by the mass media correlated with the reduced level of disturbances in daily activities in individuals with a relatively lower level of education, whereas those by the mass media and all levels of the government were found to be related in those with higher education.

Effective implementation of management policies, including health recommendations, to control the COVID-19 pandemic necessitates compliance from the public [26]. The level of compliance can depend on how individuals perceive the cost and benefits of complying with government policies since they are more likely to conform to recommendations that impose a low burden or do not interfere with their daily activities [18]. Although some changes in daily life due to the COVID-19 outbreak may be positive, such as increased family time, better work flexibility, and calmer life, many negative effects have been reported [12, 27, 28]. Specifically, social distancing measures can cause notable disturbances in daily habits, attributed to various changes in work and education related activities [9]. Quarantine measures are also known to provoke various adverse effects, including distress, anxiety, loneliness, and reduced physical activity [29]. As such, perception of the appropriateness of management strategies can affect the level of subjective disturbances in daily activities, which in turn is important in achieving public compliance and effectively implementing strategies important in managing a pandemic [30].

The significant relationship found between public perception of the appropriateness of COVID-19 management strategies implemented by the government and the level of disturbances in daily activity offers important insights because studies have shown that life satisfaction itself may not have decreased noticeably despite various government measures imposed due to the pandemic. The experience may be subjective as political distrust have been found to be associated with pandemic distress, which implies that public perception, an indirect measure of public trust, and its effect on the daily lives of individuals may be important in times of a public health crisis [31]. Transparent communication and provision of evidence-based explanation may also positively impact public perception of governmental policies [18]. Therefore, enhancing public trust may be essential to increase resilience in times of an ongoing pandemic where medical interventions may be of deficit, particularly because government trust can act as a measure of social capital [32].

The findings also reveal the potential role of mass media in causing disturbances in daily activities during an outbreak. Mass media is a major means for public health communication and acts as a source of information to the general public. The mass media serves as a platform to disseminate public health communications, provide education guidelines, and government policies implemented to combat a pandemic [19]. Mass media is also interrelated with public trust, indirectly reflected based on public perception in this study, of the government because it disseminates negative news that can not only reduce trust but also enhance political participation and increase public trust [33]. Considering the heavy reliance on mass media to disseminate public health information, the results highlight its importance in potentially lowering the level of disturbances felt or experienced by individuals during a pandemic [34].

In this study, participants who viewed that COVID-19 management measures delivered by the mass media were appropriate reported a lower level of disturbances. However, the level of disturbances in daily activities correlated with both public perception of strategies implemented by the media and the government only in individuals with higher education. This is in contrast with a previous study which concluded that the likelihood of having a positive opinion on COVID-19 response by the government was lower in those with higher education [35]. Hence, the tendencies found suggest a characteristic unique to the Korean society. Mass media may have a profound effect on the perception of daily life regardless of education level, possibly due to the need to accept new and up-to-date information during a public health crisis. The findings also infer the importance of providing accurate and unbiased social information because media coverage can affect public cognition, norms, and behaviors, and health-related beliefs [36].

This study has some limitations. First, causal inferences cannot be made assertively because this study was cross-sectional in design. Second, data were recorded annually and may not be collected at the peak of the pandemic as the Korean government declared different states of public health emergency starting from the COVID-19 outbreak. Third, although disturbances in daily activities that resulted due to the pandemic may have been positive, this could not be fully considered and reflected due to data limitation. Future studies on this topic considering the precise sense of changes perceived are needed. Last, information on public trust and interferences on daily activities related to the pandemic was based on self-reports. However, despite these limitations, this study is important because it is the first to analyze the association between public perception of the appropriateness of various COVID-19 management policies and reported levels of disturbances in daily activities using large, nationally representative data. The findings offer important insights in the development and implementation of COVID-19 related public health policies by revealing that in Korea, public perception of the appropriateness of pandemic measures were related to reported levels of daily disturbances and that such association may be important in enhancing compliance to public health measures during times of a pandemic.

In conclusion, public perception of the appropriateness of COVID-19 management policies were associated with subjective levels of daily disturbances. Specifically, public perception that measures delivered by the government and mass media were appropriate was associated with decreased levels of subjective disturbances in daily activities during the COVID-19 outbreak. The findings reveal the potential importance of public perception and minimizing its effect on the daily lives of individuals when implementing management policies during a pandemic.

## Supporting information

S1 Fig The study population selection process. (TIFF)

S2 Fig Histogram distributions of level of disturbances in daily activities. (TIFF)

S3 Fig Histogram distributions of number of public perception of management strategies. (TIFF)

S1 Table General characteristics and effect size by public perception of the type of COVID-19 management strategies. (DOCX)
