# Supplementary figures and images for: Public perception of the appropriateness of COVID-19 management strategies and level of disturbances in daily activities: A focus on educational level
Source: PLoS One. 2023 Jun 9;18(6):e0287143. doi: 10.1371/journal.pone.0287143 (PMC10256230; doi:10.1371/journal.pone.0287143)

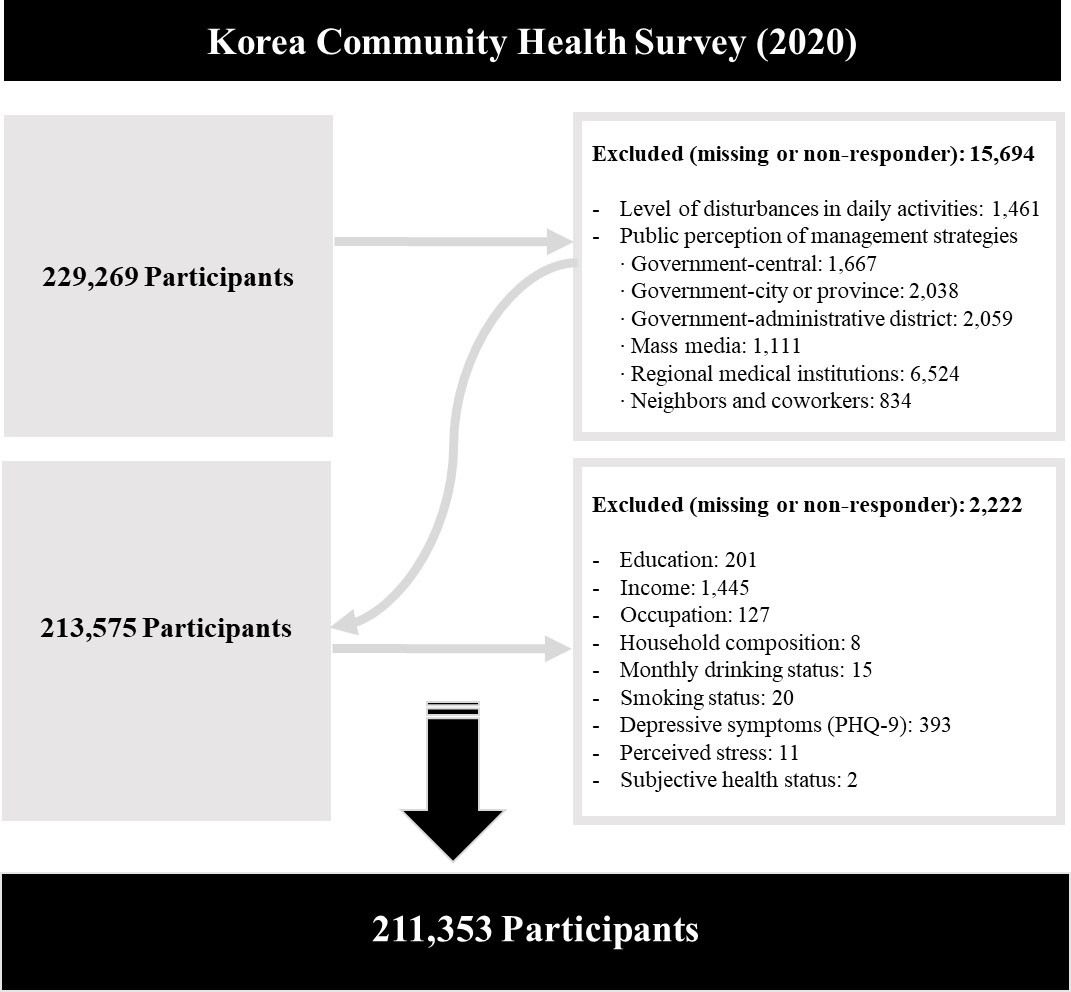

Supplement: S1 Fig — (TIFF) [file pone.0287143.s001.tiff]

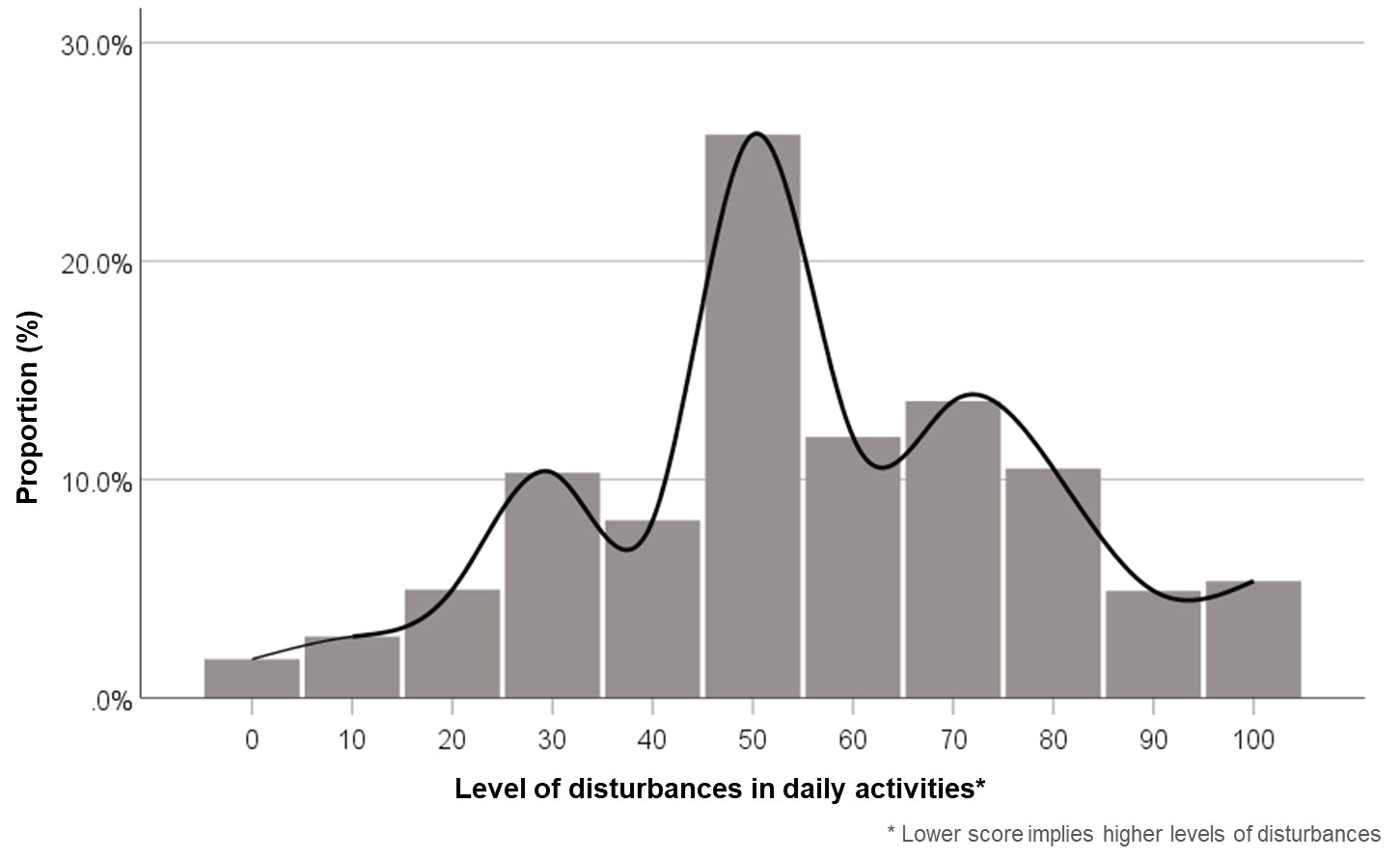

Supplement: S2 Fig — (TIFF) [file pone.0287143.s002.tiff]

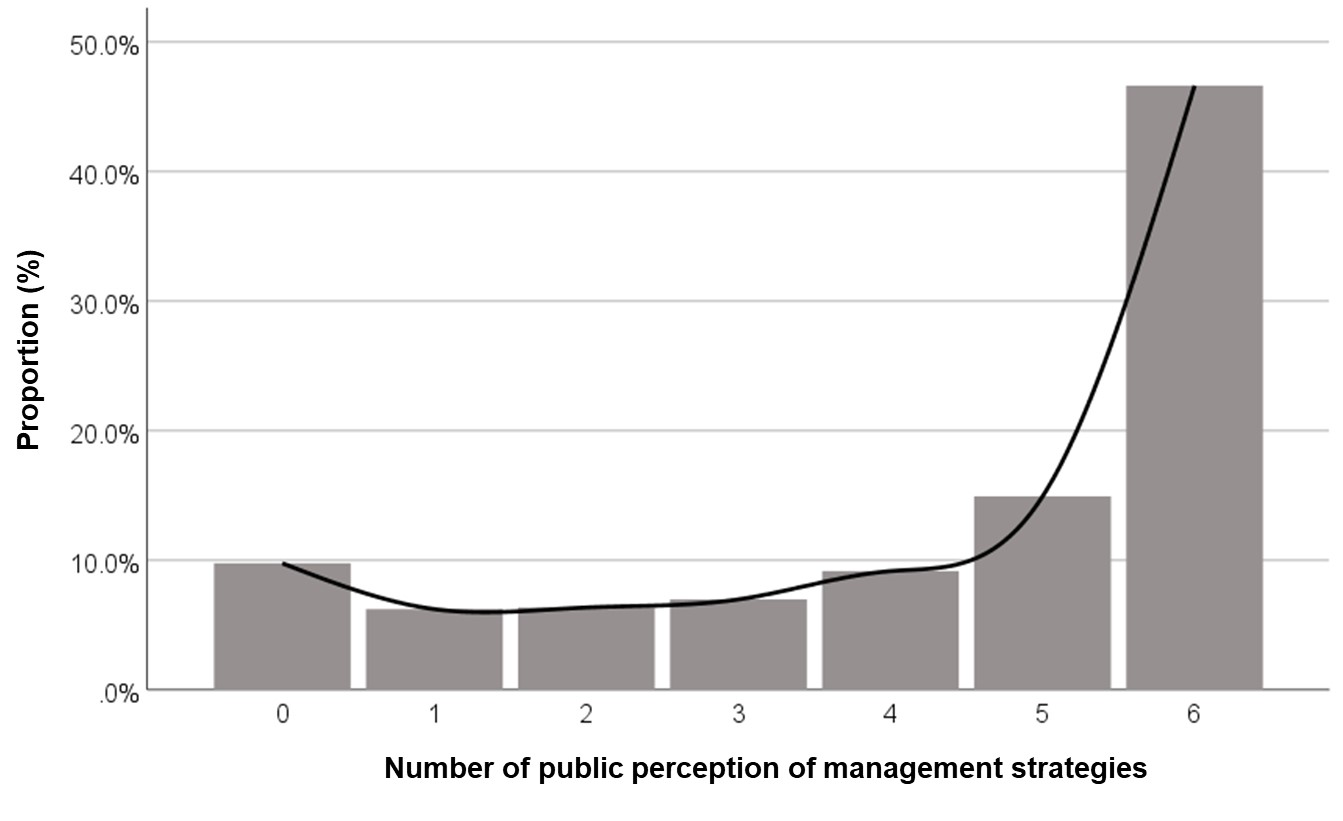

Supplement: S3 Fig — (TIFF) [file pone.0287143.s003.tiff]
