# Supplementary material for: Public perception of the appropriateness of COVID-19 management strategies and level of disturbances in daily activities: A focus on educational level
Source: PLoS One. 2023 Jun 9;18(6):e0287143. doi: 10.1371/journal.pone.0287143 (PMC10256230; doi:10.1371/journal.pone.0287143)
Supplement: S1 Table — (DOCX) [file pone.0287143.s004.docx]

| **S1 Table. General characteristics and effect size by public perception of the type of COVID-19 management strategies** | | | | | | | | | | | | | | | | | | | | | | | | | |  |  |
| --- | --- | --- | --- | --- | --- | --- | --- | --- | --- | --- | --- | --- | --- | --- | --- | --- | --- | --- | --- | --- | --- | --- | --- | --- | --- | --- | --- |
| **Variables** | | **Total** | | | | | | | | | **Level of disturbances in daily activities*** | | | | | | | | | | | | | **Effect Size (95% CI)** | |  |  |
|  |  | **N** | | | | | **%** | | | **Mean** | | | | **±** | | | **S. D** | | | ***P*-Value** | | | | **Cohen's *d*** | |  |  |
| **Public perception of management strategies** | | | |  | | |  | | |  | | | |  | | |  | |  | | | | |  |  | | |
| **Government: Central** | | | |  | | |  | | |  | | |  | | |  | | | <.001 | | | | 0.179 (0.169 , 0.189) | | | | |
| Appropriate | | | | 154,951 | | | 73.31 | | | 56.42 | | | **±** | | | 22.54 | | |  | | |  | |  | | | |
| Not appropriate | | | | 56,402 | | | 26.69 | | | 52.32 | | | **±** | | | 23.83 | | |  | | |  | |  | | | |
| **Government: City or province** | | | |  | | |  | | |  | | |  | | |  | | | <.001 | | | | | 0.206 (0.196 , 0.215) | | | |
| Appropriate | | | | 150,560 | | | 71.24 | | | 56.68 | | | **±** | | | 22.58 | | |  | | |  | |  | | | |
| Not appropriate | | | | 60,793 | | | 28.76 | | | 51.98 | | | **±** | | | 23.55 | | |  | | |  | |  | | | |
| **Government: Administrative district** | | | |  | | |  | | |  | | |  | | |  | | | <.001 | | | | | 0.221 (0.211 , 0.230) | | | |
| Appropriate | | | | 153,361 | | | 72.56 | | | 56.71 | | | **±** | | | 22.66 | | |  | | |  | |  | | | |
| Not appropriate | | | | 57,992 | | | 27.44 | | | 51.67 | | | **±** | | | 23.34 | | |  | | |  | |  | | | |
| **Mass media** | | | |  | | |  | | |  | | |  | | |  | | | <.001 | | | | | 0.178 (0.169 , 0.187) | | | |
| Appropriate | | | | 140,544 | | | 66.50 | | | 56.69 | | | **±** | | | 22.73 | | |  | | |  | |  | | | |
| Not appropriate | | | | 70,809 | | | 33.50 | | | 52.61 | | | **±** | | | 23.17 | | |  | | |  | |  | | | |
| **Regional medical institutions** | | | |  | | |  | | |  | | |  | | |  | | | <.001 | | | | | 0.168 (0.158 , 0.177) | | | |
| Appropriate | | | | 155,929 | | | 73.78 | | | 56.33 | | | **±** | | | 22.76 | | |  | | |  | |  | | | |
| Not appropriate | | | | 55,424 | | | 26.22 | | | 52.50 | | | **±** | | | 23.29 | | |  | | |  | |  | | | |
| **Neighbors and coworkers** | | | |  | | |  | | |  | | |  | | |  | | | <.001 | | | | | 0.115 (0.105 , 0.124) | | | |
| Appropriate | | | | 155,230 | | | 73.45 | | | 56.03 | | | **±** | | | 22.81 | | |  | | |  | |  | | | |
| Not appropriate | | | | 56,123 | | | 26.55 | | | 53.39 | | | **±** | | | 23.26 | | |  | | |  | |  | | | |
| **Total** | 211,353 | | | | 100.0 | | | 55.33 | | | | | ± | | | 22.96 | | |  | | | | |  |  | | |

** Lower score implies higher levels of disturbances*
